# Influence of heat stress, sex and genetic groups on reference genes stability in muscle tissue of chicken

**DOI:** 10.1371/journal.pone.0176402

**Published:** 2017-05-01

**Authors:** Haniel Cedraz de Oliveira, Antonio Amandio Pinto Garcia, Juliana Gracielle Gonzaga Gromboni, Ronaldo Vasconcelos Farias Filho, Carlos Souza do Nascimento, Amauri Arias Wenceslau

**Affiliations:** 1 Post Graduation program in Animal Science, Universidade Estadual de Santa Cruz - UESC, Ilhéus, Bahia, Brasil; 2 Departament of Rural and Animal Technology - Universidade Estadual do Sudoeste da Bahia – Campus Itapetinga – UESB, Itapetinga, Bahia, Brazil; 3 Department of Animal Science, Universidade Federal de Sergipe, São Cristovão, Sergipe, Brazil; Kumamoto University, JAPAN

## Abstract

Quantitative RT-PCR is an important technique for assessing gene expression. However, a proper normalization of reference genes prior to expression analyses of target genes is necessary. The best normalizer is that gene which remains stable in all samples from different treatments. The aim of this study was to identify stable reference genes for normalization of target genes in muscle tissue from three genetically divergent chickens groups (Peloco, Cobb 500^®^ and Caneluda) under environmental (heat stress and comfort) and sex influence. Expressions of ten reference genes were tested for stability in breast muscular tissue (*Pectoralis major* muscle). Samples were obtained from 36 males and females of two backyard breeds (Caneluda and Peloco) and one commercial line (Cobb 500^®^) under two environments. The heat stress and comfort temperature were 39 and 23°C, respectively. Animals were housed in the Animal Science Department at *Universidade Estadual do Sudoeste da Bahia*. We analyzed the expression data by four statistical tools (SLqPCR, NormFinder, Bestkeeper and Comparative CT). According to these tools, genes stability varied according to sex, genetic group and environment, however, some genes remained stable in all analyzes. There was no difference between the most stable genes for sex effect, being *MRPS27* more stable for both males and females. In general, *MRPS27* was the most stable gene. Within the three genetic groups, the most stable genes were *RPL5*, *HMBS* and *EEF1* to Cobb 500^®^, Peloco and Caneluda, respectively. Within the environment, the most stable gene under comfort and heat stress conditions was *HMBS* and *MRPS27*, respectively. BestKeeper and Comparative Ct were less correlated (28%) and SLqPCR and NormFinder were the most correlated (98%). *MRPS27*, *RPL5* and *MRPS30* genes were considered stable according the overall ranking and can be used as normalizer of relative expression of target genes in muscle tissue of chickens under heat stress.

## Introduction

Quantitative reverse transcription PCR (RT-qPCR) has been used as a reliable technique for gene expression quantification, in terms that it is more sensitive than standard PCR analysis for transcripts [1–3]. By this technique, gene quantification is obtained as the result of a gene expression of interest (target gene, TG) subtracted from the expression of a normalizer gene (reference gene, RG). Such quantitation can be very useful, but the results are highly dependent on the stability of the used RG for normalization. This procedure aims to minimize technical variations inherent to the quality and quantity of RNA introduced during the extraction procedure, reverse transcription and PCR efficiency [4]. Therefore, standardization of TG expression data is crucial to minimize technical variations that may obscure and affect the results of gene expression quantification [5].

For this technique, RG involved in basic cellular coding processes of protein such as cytoskeleton construction (actin), glycolysis (glyceraldehyde 3-phosphate dehydrogenase—GAPDH), protein folding (cyclophilin), synthesis of ribosomal subunits (rRNA), electron transport and ubiquitin degradation (ubiquitin—*UBC*) are typically used. Also, ribosomal RNA are used, such as 18S and 28S rRNA. However, an ideal RG must have stable level of expression under various experimental conditions at different stages of development, tissue type and stimuli from external environment (e.g. heat and thermal stress, immunological challenge, among others) [6].

The stability of RG can be analyzed by software that use statistical tools, such as those implemented in geNorm [7], NormFinder [8] e Bestkeeper [9] programs. These programs allow analyzing expression data obtained by RT-qPCR technique and aim to assess the stability of these genes indicating the most appropriate. Normalization procedure requires suitable amplification efficiencies of both reference and target genes [2], therefore, an appropriate choice of RG are essential. Furthermore, the use of a single RG has not been recommended, in terms that it may lead to a poor standardization [7]. According to these authors, multiple RG should be used for proper gene expression quantification. Recently, the use of multiple RG has been used and recommended as a suitable approach for TG normalization in chicken [10].

Several studies have indicated suitable RG in cattle [11], pigs [12], ovine [13], goats [14], horse [15] and fish [16,17]. Also, studies have been carried out to identify suitable RG in chickens (*Gallus gallus*) [10,18]. However, to date, there are no studies reported that have evaluated suitable RG as normalizer under similar conditions as proposed in this study (genetic group, sex and environment).

Considering the above, we aimed to identify stable reference genes for normalization of target genes in muscle tissue from three genetically divergent groups of chicken (Peloco, Caneluda and Cobb 500^®^) considering the influence of environment (heat stress and comfort) and sex.

## Material and methods

### Ethical approval

Experiment procedures were approved by the Ethics Committee on Animal Use—CEUA of Universidade Estadual do Sudoeste da Bahia (UESB), protocol 109/2015.

### Animals

In this study, we used 36 males and females birds, being 12 chicks of each breed (Peloco and Caneluda, backyard breeds, and Cobb 500^®^, commercial line). Commercial animals were purchased a week after the birth of backyard birds in the Universidade Estadual do Sudoeste da Bahia (UESB), Itapetinga Campus, and raised under the same environmental conditions from November 2 to December 2 of 2015 with an average temperature of 26.5°C. The predominant climate of Itapetinga region is the semi-arid, in which the temperature increases during the day and decrease during the night. Nutritional diets followed the requirements of the Brazilian Tables for Poultry and Swine [19] and the feed was produced in the poultry sector of UESB (Table 1). All birds were raised in semi-open stalls and lined with wood shavings (wood chips).

**Table 1:** Initial feed used in the production of chicks up to 30 days of age (ROSTAGNO, GOMES, 2011).

Corn	61.1%
Soybean Meal	35.0%
Dicalcium Phosphate	2.00%
Limestone	1.10%
NaCl	0.30%
Vitamin And Mineral Supplement	0.40%
Nutritional Levels	
Crude Protein	21.2%
Metabolizable Energy	2.89%
Calcium	1.01%
Phosphor Available	0.49%
Sodium	1.63%
Lysine	1.10%
Methionine + Cysteine	0.74%

### Heat stress

Heat stress was provided in two stages so that all birds had the same slaughter age (30 days). First, six birds of Peloco breed and six birds of Caneluda breed were subjected to heat stress under an average temperature of 39.5°C and environmental relative humidity of 60% for one hour. In the second stage, six birds of Cobb 500^®^ line were subjected to heat stress with the same conditions of temperature and humidity for 30 minutes. During the heat stress period, animals had *ad libitum* access to food and water.

During the heat stress, birds were constantly observed for behavioral changes, in order to avoid deaths caused by excessive temperature. The acute heat stress was determined at the moment that most of the birds (±90%) were prostrate (lying with the abdominal faced down), and with increased respiratory rate. All birds were slaughtered by cervical dislocation after heat stress period.

All control birds (six birds of each genetic group) were slaughtered by cervical dislocation at the second stage of the experiment at 4 am (local time) to ensure thermal comfort temperature (23°C).

### Tissue sampling

After slaughter, muscle tissue samples were collected from the breast (*Pectoralis major*), placed in cryogenic tubes, identified and stored in liquid nitrogen. Samples were transported to the Veterinary Genetics Laboratory of the Universidade Estadual de Santa Cruz (UESC), separated and stored in Ultrafreezer (-80°C).

### Extraction, quantification and quality of total RNA

For total RNA extraction, we used the commercial kit SV Total RNA Isolation System^®^ (Promega Corporation, Madison, USA) according to manufacturer's protocol. The concentration and quality of RNA were verified by NanoDrop 2000 spectrophotometer (Thermo Fisher Scientific Inc, Carlsbad, CA, USA) using the absorbance at 230, 260, 280nm. Besides, RNA integrity were analyzed by the presence of bands corresponding to 28S and 18S ribosomal RNAs observed through electrophoresis of 1 ug of RNA in 1% agarose gel stained with ethidium bromide.

### Reverse transcription of mRNA

The commercial kit GoScript TM Reverse Transcription System (Promega Corporation, Madison, USA) was used for reverse transcription of mRNA. Five micrograms of total RNA from muscle samples were mixed to 1μl of Oligo(dT) (500μg/ml) and heated in 70°C for 5 minutes. After incubation, 4μl of 5X Reaction Buffer, 3.2μl MgCl_2_, 1μl dNTP (0,5mM), 1μl of reverse transcriptase enzyme, 0.5μl of inhibitor of recombinant ribonuclease RNaseOUT (20units) and ultrapure water completing 15μl. This mix were added to RNA+OligodT mix completing a volume total of 20μl and incubated on a thermocycler. Anneal at 25°C for 5 minutes; extend at 42°C for one hour, and 70°C for 15 minutes to inactivate the reverse transcriptase. After reverse transcription, ultrapure water was added in each tube to complete a final volume of 100 ul and stored at -20°C. The concentration of cDNA was measured by NanoDrop 2000 spectrophotometer (Thermo Fisher Scientific Inc, Carlsbad, CA, USA) using the absorbance at 230, 260, 280nm.

### Reference genes selection and RT-qPCR optimization

Sequences of ten reference genes were selected from literature [18] and used for analysis of expression stability using RT-qPCR (Table 2). To obtain the standard curve, we used a cDNA pool of all treatments aiming to optimize and calculate the PCR efficiency. We used three cDNA concentrations (15, 45 and 135ng/μl) and three primer concentrations (200, 400, 800 mM).

**Table 2:** Description of *Gallus gallus* reference genes, their specific primers used in RT-qPCR analysis and parameters derived from RT-qPCR analysis. All primers were designed by NASCIMENTO et al., (2015).

GENE	GENE ID	SEQUENCE (5'-3')	DESCRIPTION	LENGTH (PB)
***ACTA1***	ENSGALE00000120039	F: CTCCGGCGATGGTGTGA	Actin, Alpha 1, Skeletal Muscle	122
		R: CAGTCAGGATCTTCATCAGGTAGT		
***UBC***	M11100.1	F: CACCCTGTCTGACTACAACATC	Ubiquitin C	92
		R: ACAAGACTGCTGACAACAACTA		
***HPRT1***	AJ132697	F: GCACTATGACTCTACCGACTATTG	Hypoxanthine Phosphoribosyltransferase 1	112
		R: CAGTTCTGGGTTGATGAGGTT		
***LDHA***	ENSGALE00000067556	F: CTATGTGGCCTGGAAGATCAG	Lactate dehydrogenase A	124
		R: GCAGCTCAGAGGATGGATG		
***EEF1***	NM_204157.2	F: GCCCGAAGTTCCTGAAATCT	Eukaryotic translation elongation factor 1 alpha 2	102
		R: AACGACCCAGAGGAGGATAA		
***MRPS27***	XM_424803	F: GCTCCCAGCTCTATGGTTATG	Mitochondrial ribosomal protein S27	124
		R: ATCACCTGCAAGGCTCTATTT		
***MRPS30***	NM_204939.1	F: CCTGAATCCCGAGGTTAACTATT	Mitochondrial ribosomal protein S30	107
		R: GAGGTGCGGCTTATCATCTATC		
***RPL5***	NM_204581.4	F: AATATAACGCCTGATGGGATGG	Ribosomal protein L5	99
		R: CTTGACTTCTCTCTTGGGTTTCT		
***TFRC***	ENSGALE00000080099	F: CTCCTTTGAGGCTGGTGAG	Transferrin receptor (p90, CD71)	89
		R: CGTTCCACACTTTATCCAAGAAG		
***HMBS***	ENSGALE00000001922	F: TGACCTGGTAGTTCACTCCTT	Hydroxymethylbilane synthase	75
		R: TTGCAAATAGCACCAATGGTAAAG		

RT-qPCR reaction conditions were set with initial denaturation temperature at 95°C for two minutes, and 40 cycles of denaturation at 95°C for 15 seconds. The extension temperature was individually standardized for each pair of primer for 60 seconds. At the end of amplification reaction, we included an additional step with gradual temperature increasing from 60 to 95°C for dissociation curve analysis. Amplification of all genes was performed in duplicate in a 7500 Fast Real Time PCR System (Applied Biosystems, Foster City, CA, USA). Results were obtained by using the Sequence Detection Systems software (V. 2.0.6) (Applied Biosystems Foster City, CA, USA) that generated the cycle threshold (Ct) parameter. The Ct values of duplicates were obtained directly from the above program and used to calculate the average Ct and standard deviation. PCR amplification efficiency was calculated for each reference gene using the following formula: E = (10^^(-1/slope)-1^) x 100 [20]. After efficiency analysis, the most appropriate annealing temperature and primer concentration were used in PCR reactions.

### Real time quantitative PCR

The RT-qPCR reaction was performed using SYBR Green detection kit with GoTaq qPCR Master Mix (Promega, Madison, WI, EUA), using specific primers. Gene amplification was performed in duplicate using the Real Time PCR 7500 Fast system (Applied Biosystems, Foster City, CA, EUA) and the results obtained with the *Sequence Detection Systems* program (V. 2.0.6) (Applied Biosystems, Foster City, CA, EUA) that generated the *cycle threshold* (Ct) parameters.

The Ct values were exported to Microsoft Excel to calculate the Ct mean, standard deviation and the standard curve for each gene. A negative control (ultra-pure water) also was added in each assay. The qPCR reaction conditions were defined as follow: Initial denaturation at 95°C during ten minutes and 40 cycles of denaturation at 95°C for 15 seconds. The extension temperature between 60 and 64°C during one minute was ideal for all primers. Ct values of control wells were excluded from subsequent analyzes as well as the undetectable values.

### Determination of expression stability of reference genes

The average Ct values of reference genes were used to set the input files according to each software. To calculate the stability of reference genes, four different analysis methods were used: SLqPCR [21], NormFinder [8], Comparative Ct (ΔCt) [22], and BestKeeper [9]. All analyzes were performed in the statistical environment R [23], except Bestkeeper, which was analyzed in Microsoft Excel. Analyses were performed according to authors’ recommendations of each tool.

The SLqPCR is a statistical analysis package for R software [23] using the method of VANDESOMPELE et al. (2002). To perform the stability analysis, it is necessary to transform Ct values in relative values (Q). This method automatically calculates a measure (M) of expression stability for each control gene in a given group of samples. The lower the M value, more stable, and thus recommending a pair of RG as normalizer.

The NormFinder was used as an algorithm for R statistical environment developed by Andersen et al., (2004) and provides information on intra and inter-group variability indicating the best endogenous control [8]. This algorithm indicates the expression of the most stable gene by the lower mean values of stability (S), identifying the reference genes according to their groups of inter and intra-expression variation, obtaining a single gene with a stable expression.

BestKeeper analyzes the variability of reference genes expression through the Ct calculation [24]. It uses a comparative Ct that calculates gene expression changes based on relative difference between an experimental sample and normalizer [25]. Data interpretation is based on standard deviation [±CP] that should be less than 1, on standard deviation [± X-Fold] being less than 2 and coefficient of correlation [r], which should be as large as possible [9]. The correlation coefficient (r) and standard deviation (SD) are the main parameters to evaluate the expression of reference genes and must be large and low, respectively [9]. Furthermore, authors suggest excluding genes that have SD above 1.5. They do not indicate the importance of each measurement, leaving to researcher's criteria the decision to choose which parameter will be used. Based on these previous studies, it was decided to use only the SD to select the most stable RG.

The method of Comparative Ct (ΔCt) determines the most stable gene by comparison of relative expression of pairs of genes in each sample of the treatment [22]. The stable reference genes are those that remain constant ΔCt values for all tested samples. For each tool, a ranking of stability was obtained.

RankAggreg package [26] was used in R software by Monte Carlo algorithm to calculate the Spearman distance and obtain the overall ranking. This package sorts from the most stable to the less stable genes, considering the stability values and the frequency that each gene is displayed according to the algorithms of stability analysis tools (SLqPCR, NormFinder, BestKeeper and Comparative Ct).

Ct data was converted to linear values (2^-ct^) as recommended by LIVAK and SCHMITTGEN [27]. ANOVA was performed to identify the difference among effects and their interactions (P≤0.05).

## Results

### Efficiency and specificity of primers

**Fig 1:**
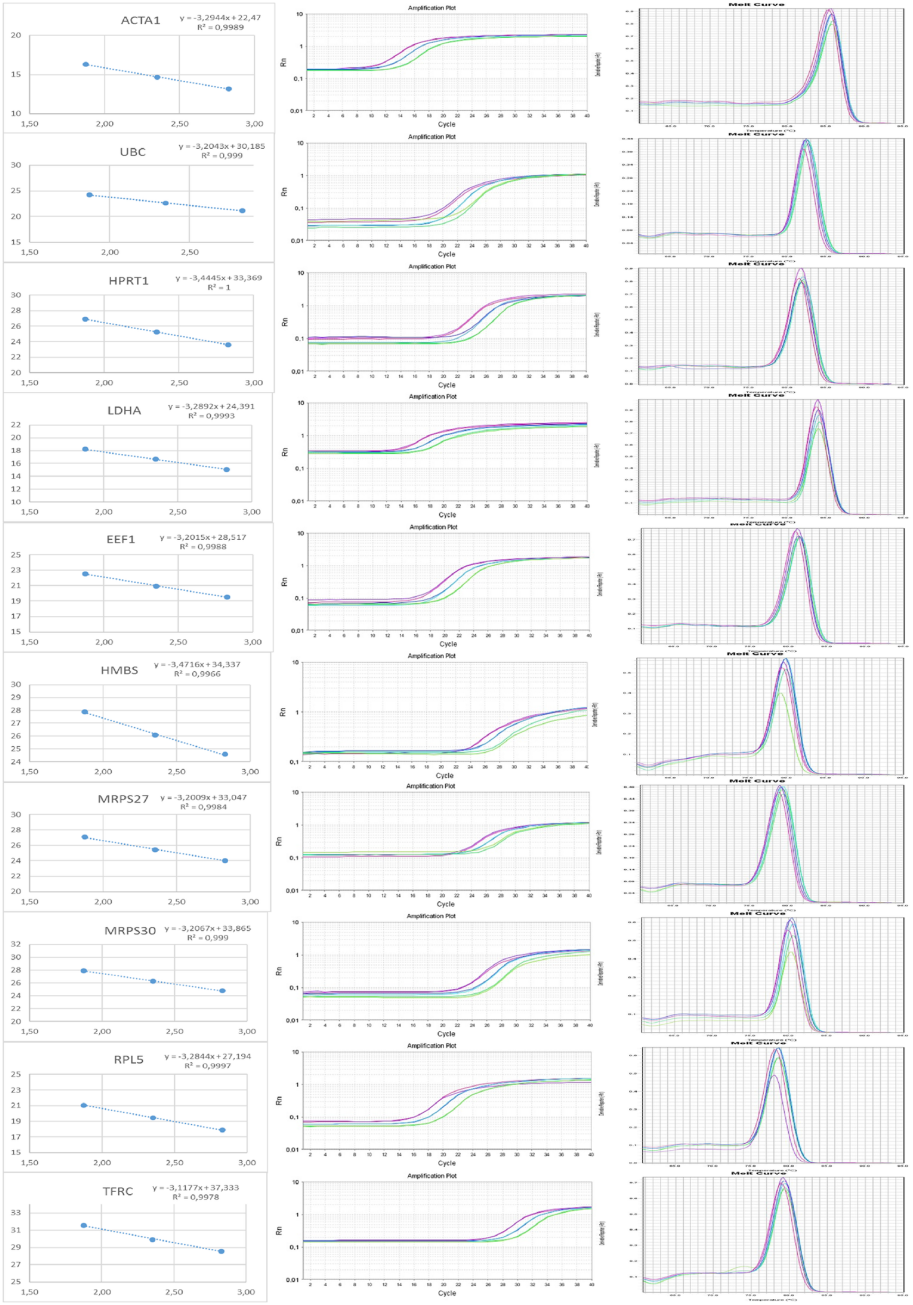
Curves of regression, amplification and dissociation from efficiency test of 10 reference genes of chicken in RT-qPCR reactions. All dissociation curves showed only one peak.

To validate the reference genes used in this study, we tested primers efficiency to check their main features prior to RT-qPCR. The annealing temperature of primers ranged from 60 to 64°C and RG expression efficiency varied between 94 and 109% corresponding to a slope of -3.25 to -3.11. The coefficient of determination values (R^2^) were greater than 0.99 (Table 3). Primers specificity was assessed by dissociation curve, which showed only one peak indicating that no primer dimer was detected, presenting excellent performance (Fig 1).

**Table 3:** Parameters of reference genes primers specific for chickens obtained from efficiency curve analysis of RT-qPCR.

GENE	AT (°C)	[CDNA]	[PRIMER]	EFICIENCY (%)	R^2^	SLOPE
***ACTA1***	60	45ng/μl	800mM	101	0.999	-3.294
***UBC***	60	45ng/μl	800mM	105	0.999	-3.294
***HPRT1***	60	45ng/μl	800mM	95	1	-3.445
***LDHA***	60	45ng/μl	800mM	101	0.999	-3.289
***EEF1***	62	45ng/μl	800mM	105	0.999	-3.202
***MRPS27***	62	45ng/μl	800mM	105	0.998	-3.201
***MRPS30***	62	45ng/μl	800mM	105	0.999	-3.207
***RPL5***	62	45ng/μl	800mM	102	1.000	-3.284
***TFRC***	62	45ng/μl	400mM	109	0.998	-3.118
***HMBS***	64	45ng/μl	800mM	94	0.996	-3.254

AT = Annealing temperature; SLOPE = Slope of the line; R^2^ = coefficient of determination; [CDNA] = cDNA concentration; [PRIMER] = Primer Concentration

### Descriptive statistics of reference genes

Ct values < 29 are strong positive reactions indicating abundance of nucleic acid in the sample, Cts of 30–37 are positive reactions indicating moderate amounts of nucleic acid, Cts of 38–40 are weak reactions indicating minimal amounts of nucleic acid [28].

Ten reference genes were analyzed by RT-qPCR. It is clear to note that there is an expression variability among the quantification cycles of the 10 genes. Based on their expression, these genes were grouped into two categories (strong, moderate). Nine genes (*ACTA1*, *LDHA*, *EEF1*, *RPL5*, *UBC*, *HPRT1*, *MRPS27*, *HMBS*, *MRPS30*,) showed strong expression with Ct values ranging between 12 and 29 cycles, and only one gene (*TFRC*) presented moderate expression, ranging from 25 to 35 cycles. Bestkeeper was used to obtain the range of variation coefficient. The lowest value was observed for *MRPS27* gene (CV = 3.2%) and the highest for *ACTA1* (CV = 7.8%), as showed on Table 4.

**Table 4:** Descriptive statistics of expression levels of reference genes for chickens obtained by BestKeeper (n = 36).

n = 36	*ACTA1*	*UBC*	*HPRT1*	*LDHA*	*MRPS27*	*EEF1*	*HMBS*	*MRPS30*	*RPL5*	*TFRC*
geo Mean [Ct]	14.94	22.84	25.25	17.28	25.54	21.42	24.85	26.87	19.71	28.67
ar Mean [Ct]	15.02	22.89	25.27	17.34	25.56	21.45	24.87	26.89	19.73	28.74
min [Ct]	12.23	20.31	23.21	14.83	23.09	19.23	21.28	24.27	17.89	25.5
max [Ct]	21.54	27.18	27.97	22.38	28.91	24.09	26.7	29.88	22.66	35.8
**std dev [± Ct]**	**1.176**	**1.167**	**0.832**	**1.101**	**0.82**	**0.927**	**0.836**	**0.919**	**0.666**	**1.458**
**CV [% Ct]**	**7.829**	**5.098**	**3.291**	**6.35**	**3.206**	**4.32**	**3.361**	**3.419**	**3.374**	**5.075**
coeff. of corr. [r]	0.819	0.796	0.865	0.815	0.786	0.727	0.365	0.821	0.794	0.664
p-value	0.001	0.001	0.001	0.001	0.001	0.001	0.028	0.001	0.001	0.001

Abbreviations: Ct: Cycle threshold; geo Mean [Ct]: Geometric mean of Ct; ar Mean [Ct]: Arithmetic mean of Ct; Min [Ct] and Max [Ct]: Cycle threshold values; **std dev [± Ct]** [±Ct]: Standard deviation (SD) of Ct; **CV [% Ct]**: Coefficient of variation of Ct levels in percentage; coeff. of corr. [r]: Coefficient of correlation; SD and CV are indicated in bold.

### Stability analysis of reference genes expression

Aiming to cover all factors on stability analysis, data were divided into 4 groups of analysis: General group (not considering differences between genetic groups, environment and sex), Genetic group (considering the backyard breeds and commercial line), Environment (acute heat stress and thermal comfort) and Sex (male and female).

#### SLqPCR

According to this tool, the best reference genes for each treatment were: General, *MRPS27*/*MRPS30* (M = 0.34); Genetic group: Peloco and Cobb 500^®^, *MRPS27*/*MRPS30* (M = 0.25 and M = 0.35, respectively), Caneluda, *HPRT1*/*HMBS* (M = 0.36); Environment: Heat stress, *MRPS27*/*MRPS30* (M = 0.40) and Comfort *ACTA1*/*LDHA* (M = 0.39); Sex: male and female, *MRPS27*/*MRPS30*, M = 0.26, M = 0.40, respectively). The less stable gene was *TFRC* for all groups except in Genetic group for Cobb 500^®^, which was the *ACTA1* (Table 5).

**Table 5:** Ranking with stability values for each treatment (genetic group, sex, and environment) in chicken obtained from SLqPCR package. Values into the parenthesis refer to ranking by SLqPCR to each treatment. Values on the last column refer to the ranking position by RankAggreg package among all the treatments.

SLqPCR Package									
GENES	General (n = 36)	Peloco (n = 12)	Cobb (n = 12)	Caneluda (n = 12)	Comfort (n = 18)	Stress (n = 18)	Male (n = 18)	Female (n = 18)	Rank
***ACTA1***	1.05 (7)	0.50 (4)	1.54 (10)	0.59 (7)	**0.39 (1)**	1.13 (7)	1.4 (8)	0.58 (4)	6
***UBC***	1.14 (8)	0.65 (8)	1.13 (7)	0.76 (9)	0.55 (4)	1.24 (8)	1.51 (9)	0.66 (7)	9
***HPRT1***	0.81 (5)	0.54 (6)	1.22 (8)	**0.36 (1)**	**0.44 (3)**	0.63 (4)	0.96 (5)	0.63 (6)	5
***LDHA***	0.98 (6)	0.52 (5)	1.39 (9)	0.68 (8)	**0.39 (1)**	1.01 (6)	1.30 (7)	0.7 (8)	7
***MRPS27***	**0.34 (1)**	**0.25 (1)**	**0.35 (1)**	0.47 (4)	0.92 (9)	**0.40 (1)**	**0.26 (1)**	**0.40 (1)**	1
***EEF1***	0.66 (4)	0.58 (7)	0.73 (4)	**0.42 (3)**	0.78 (6)	0.81 (5)	0.73 (4)	0.60 (5)	4
***HMBS***	1.21 (9)	0.93 (9)	0.91 (5)	**0.36 (1)**	0.61 (5)	1.38 (9)	1.14 (6)	0.81 (9)	8
***MRPS30***	**0.34 (1)**	**0.25 (1)**	**0.35 (1)**	0.53 (6)	0.9 (8)	**0.40 (1)**	**0.26 (1)**	**0.40 (1)**	2
***RPL5***	**0.49 (3)**	**0.42 (3)**	**0.51 (3)**	0.49 (5)	0.84 (7)	**0.51 (3)**	**0.49 (3)**	**0.49 (3)**	3
***TFRC***	1.35 (10)	1.21 (10)	1.03 (6)	0.92 (10)	1.08 (10)	1.51 (10)	1.63 (10)	1.00 (10)	10

This analysis method does not differentiate between positions 1 and 2 (the two most stable genes have the same stability value).

#### NormFinder

The most stable genes pointed by NormFinder for each group were: General, *RPL5* (S = 0.43); Genetic group: Peloco, *LDHA* (S = 0.19), Cobb 500^®^, *HMBS* (S = 0.22) and Caneluda, *EEF1* (S = 0.15); Environment: Heat stress, *MRPS27* (S = 0.26) and Comfort, *HMBS* (S = 0.17); Sex: male, *MRPS27* (S = 0.17) and female, *MRPS30* (S = 0.21) as observed on Table 6.

**Table 6:** Ranking with stability values for each treatment (genetic group, sex, and environment) in chicken obtained from NormFinder. Values into the parenthesis refer to ranking by Normfinder to each treatment. Values on the last column refer to the ranking position by RankAggreg package among all the treatments.

Normfinder (R)									
GENE	General (n = 36)	Peloco (n = 12)	Cobb (n = 12)	Caneluda (n = 12)	Comfort (n = 18)	Stress (n = 18)	Male (n = 18)	Female (n = 18)	Rank
***ACTA1***	0.61 (7)	**0.25 (3)**	0.83 (10)	0.27 (6)	0.26 (7)	0.66 (7)	**0.26 (3)**	0.48 (9)	7
***UBC***	0.60 (6)	0.58 (8)	0.53 (6)	0.38 (9)	0.20 (4)	0.74 (8)	0.45 (8)	0.27 (5)	8
***HPRT1***	**0.46 (2)**	0.34 (5)	0.46 (4)	**0.19 (2)**	**0.20 (3)**	0.46 (4)	0.33 (7)	0.22 (4)	4
***LDHA***	0.62 (8)	**0.19 (1)**	0.70 (9)	0.37 (8)	0.25 (6)	0.62 (6)	0.29 (4)	0.47 (8)	6
***MRPS27***	**0.49 (3)**	0.36 (6)	**0.45 (3)**	0.21 (4)	0.29 (8)	**0.26 (1)**	**0.17 (1)**	**0.22 (3)**	1
***EEF1***	0.49 (4)	0.47 (7)	0.6 (8)	**0.15 (1)**	0.23 (5)	0.54 (5)	0.30 (5)	0.28 (6)	5
***HMBS***	0.70 (9)	1.15 (9)	**0.22 (1)**	**0.20 (3)**	**0.17 (1)**	0.90 (10)	0.55 (9)	0.38 (7)	9
***MRPS30***	0.50 (5)	0.30 (4)	0.50 (5)	0.23 (5)	0.29 (9)	**0.30 (2)**	**0.25 (2)**	**0.21 (1)**	3
***RPL5***	**0.43 (1)**	**0.21 (2)**	**0.37 (2)**	0.28 (7)	**0.19 (2)**	**0.33 (3)**	0.31 (6)	**0.21 (2)**	2
***TFRC***	1.00 (10)	1.15 (10)	0.56 (7)	0.6 (10)	0.59 (10)	0.88 (9)	0.61 (10)	0.65 (10)	10

#### BestKeeper

*TFRC* and *ACTA1* genes obtained SD greater than 1.5 and were excluded from analysis. The most stable gene recommended by BestKeeper for each group was: General, *RPL5* (SD = 0.67); Genetic group: Peloco, *RPL5* (SD = 0.44), Cobb 500^®^, *HMBS* (SD = 0.48) and Caneluda, *RPL5* (SD = 0.37); Environment: Heat Stress, *RPL5* (SD = 0.54) and Comfort, *HMBS* (SD = 0.63); Sex: Male, *RPL5* (SD = 0.69) and Female, *MRPS27* (SD = 0.61) as observed on Table 7.

**Table 7:** Ranking with stability values for each treatment (genetic group, sex, and environment) in chicken obtained from BestKeeper tool. Values into the parenthesis refer to ranking by Bestkeeper to each treatment. Values on the last column refer to the ranking position by RankAggreg package among all the treatments.

BestKeeper (SD)									
GENES	General (n = 36)	Peloco (n = 12)	Cobb (n = 12)	Caneluda (n = 12)	Comfort (n = 18)	Stress (n = 18)	Male (n = 18)	Female (n = 18)	Rank
***ACTA1***	1.18 (9)	0.67 (6)	1.52 (10)	0.87 (8)	1.03 (8)	1.37 (9)	1.34 (8)	1.06 (9)	9
***UBC***	1.17 (8)	0.91 (8)	**0.69 (2)**	0.84 (7)	1.06 (9)	1.26 (7)	1.46 (9)	0.87 (7)	7
***HPRT1***	**0.83 (3)**	0.48 (4)	1.05 (6)	0.54 (5)	**0.80 (3)**	0.91 (5)	0.94 (4)	0.73 (4)	4
***LDHA***	1.10 (7)	0.66 (5)	1.39 (9)	1.01 (9)	0.91 (4)	1.35 (8)	1.21 (7)	0.99 (8)	8
***MRPS27***	**0.82 (2)**	**0.44 (2)**	1.06 (7)	**0.50 (3)**	0.98 (7)	**0.65 (2)**	**0.87 (3)**	**0.61 (1)**	2
***EEF1***	0.93 (6)	0.80 (7)	**0.84 (3)**	**0.47 (2)**	0.97 (5)	0.89 (4)	1.04 (5)	0.86 (6)	6
***HMBS***	0.84 (4)	1.40 (9)	**0.48 (1)**	0.52 (4)	**0.63 (1)**	1.04 (6)	**0.84 (2)**	0.79 (5)	3
***MRPS30***	0.92 (5)	**0.47 (3)**	1.13 (8)	0.63 (6)	0.98 (6)	**0.86 (3)**	1.08 (6)	**0.69 (3)**	5
***RPL5***	**0.67 (1)**	**0.44 (1)**	0.87 (4)	**0.37 (1)**	**0.79 (2)**	**0.54 (1)**	**0.69 (1)**	**0.66 (2)**	1
***TFRC***	1.46 (10)	1.68 (10)	1.04 (5)	1.39 (10)	1.32 (10)	1.37 (10)	1.62 (10)	1.22 (10)	10

#### Comparative Ct (ΔCt)

The most stable gene pointed by Comparative Ct analysis for each group was: General, *ACTA1* (SD = 0.14); Genetic group: Peloco, *ACTA1* (SD = 0.16), Cobb 500^®^, *MRPS27* (SD = 0.18) and Caneluda *HMBS* (SD = 0.14); Environment: Heat stress, *ACTA1* (SD = 0.20) and Comfort, *MRPS27* (SD = 0.14); Sex: male, *ACTA1* (SD = 0.10) and female, *MRPS27* (SD = 0.25) as observed on Table 8.

**Table 8:** Ranking with stability values for each treatment (genetic group, sex, and environment) in chicken obtained from Comparative Ct (ΔCt) analysis. Values into the parenthesis refer to ranking by Comparative Ct (ΔCt) to each treatment. Values on the last column refer to the ranking position by RankAggreg package among all the treatments.

Comparative Ct (ΔCT)									
GENES	General (n = 36)	Peloco (n = 12)	Cobb (n = 12)	Caneluda (n = 12)	Comfort (n = 18)	Stress (n = 18)	Male (n = 18)	Female (n = 18)	Rank
***ACTA1***	**0.14 (1)**	**0.16 (1)**	0.19 (4)	0.17 (6)	**0.15 (3)**	**0.20 (1)**	**0.10 (1)**	0.26 (5)	1
***UBC***	0.18 (7)	0.27 (8)	0.27 (8)	0.27 (9)	0.27 (9)	0.27 (8)	0.27 (9)	0.27 (6)	9
***HPRT1***	**0.15 (3)**	0.19 (6)	**0.19 (2)**	0.16 (4)	**0.14 (2)**	0.22 (4)	**0.11 (3)**	**0.26 (3)**	4
***LDHA***	0.16 (5)	**0.17 (2)**	0.20 (6)	0.20 (7)	0.15 (4)	0.25 (6)	0.11 (4)	0.31 (8)	6
***MRPS27***	**0.15 (2)**	0.19 (7)	**0.18 (1)**	**0.15 (3)**	**0.14 (1)**	**0.20 (2)**	**0.11 (2)**	0.26 (4)	2
***EEF1***	0.20 (8)	**0.18 (3)**	0.31 (10)	0.17 (5)	0.15 (6)	0.26 (7)	0.15 (6)	0.30 (7)	7
***HMBS***	0.27 (10)	0.37 (9)	0.20 (5)	**0.14 (1)**	0.16 (8)	0.36 (10)	0.28 (10)	0.35 (10)	8
***MRPS30***	0.16 (4)	0.19 (5)	0.20 (7)	**0.15 (2)**	0.15 (7)	**0.21 (3)**	0.12 (5)	**0.25 (1)**	3
***RPL5***	0.16(6)	0.19 (4)	**0.19 (3)**	0.23 (8)	0.15 (5)	0.22 (5)	0.12 (6)	**0.25 (2)**	5
***TFRC***	0.24 (9)	0.39 (10)	0.29 (9)	0.28 (10)	0.27 (10)	0.29 (9)	0.20 (8)	0.31 (9)	10

### Spearman correlation between tools

In order to verify the correlation between tools, Spearman's rank correlation analysis was used by SAS^®^ software. Tools were compared by the general order of reference genes. The difference was considered significant if P≤0.05. SLqPCR and NormFinder were the most correlated tools, with 95% of similarity, whereas the Comparative Ct and Bestkeeper were less correlated with only 28% of similarity. For NormFinder and BestKeeper, and NormFinder and Comparative Ct, moderate correlation (68%) for both combinations was observed, while SLqPCR and BestKeeper obtained a correlation of 65%. Correlation between SLqPCR and Comparative Ct was moderate with 75% of similarity (Table 9).

**Table 9:** Spearman correlation comparing all tools based on the average ordering of reference genes stability.

	BestKeeper	SLqPCR	NormFinder	Ct Comparativo
BestKeeper	1			
SLqPCR	0.65	1		
	**0.0425**			
NormFinder	0.68	0.95	1	
	**0.0289**	**<.0001**		
Comparative Ct	0.28	0.75	0.68	1
	**0.425**	**0.0133**	**0.0289**	

P-value is written in bold.

### Overall rank of reference genes

The overall ranking of the most stable RG obtained by this package is observed on Table 10.

**Table 10:** Overall ranking of reference genes in chickens obtained by different tools (SLqPCR, NormFinder, Bestkeeper and Comparative Ct) and ranked by RankAggreg package according to each treatment (genetic groups, environment and sex).

GENERAL	PELOCO	COBB	CANELUDA	COMFORT	STRESS	MALE	FEMALE	Overall Spearman	Ranking
*MRPS27*	*RPL5*	*HMBS*	*EEF1*	*HMBS*	*MRPS27*	*MRPS27*	*MRPS27*	***MRPS27***	1
*RPL5*	*MRPS30*	*RPL5*	*HMBS*	*HPRT1*	*RPL5*	*MRPS30*	*MRPS30*	***RPL5***	2
*MRPS30*	*MRPS27*	*MRPS27*	*HPRT1*	*LDHA*	*MRPS30*	*RPL5*	*RPL5*	***MRPS30***	3
*HPRT1*	*LDHA*	*MRPS30*	*MRPS27*	*RPL5*	*HPRT1*	*HPRT1*	*HPRT1*	***HPRT1***	4
*EEF1*	*ACTA1*	*HPRT1*	*MRPS30*	*ACTA1*	*EEF1*	*EEF1*	*UBC*	***EEF1***	5
*ACTA1*	*HPRT1*	*UBC*	*RPL5*	*EEF1*	*LDHA*	*ACTA1*	*EEF1*	***ACTA1***	6
*LDHA*	*EEF1*	*TFRC*	*ACTA1*	*MRPS27*	*ACTA1*	*LDHA*	*HMBS*	***LDHA***	7
*UBC*	*UBC*	*EEF1*	*LDHA*	*MRPS30*	*UBC*	*HMBS*	*ACTA1*	***UBC***	8
*HMBS*	*HMBS*	*LDHA*	*UBC*	*UBC*	*TFRC*	*UBC*	*LDHA*	***HMBS***	9
*TFRC*	*TFRC*	*ACTA1*	*TFRC*	*TFRC*	*HMBS*	*TFRC*	*TFRC*	***TFRC***	10

*RPL5* gene expression was statistically different only between genetic groups (P = 0.0294). Nevertheless, *MRPS30* and *MRPS27* were not different among the studied effects. Interactions were not statistically significantly. According to these results, the normalization factor was calculated as the geometric mean of *MRPS30*, *MRPS27* and *RPL5* genes. The normalization factor was not different between breed, treatment or sex (Table 11).

**Table 11:** ANOVA analysis of Ct values of genetic group, sex and environment effects among previous chosen genes.

	Genetic Group		Sex		Environment	
	F-value	P-value	F-value	P-value	F-value	P-value
*ACTA1*	4.630	0.020	0.924	0.346	0.189	0.667
*UBC*	10.254	0.001	1.211	0.282	0.943	0.341
*HPRT1*	4.662	0.020	1.421	0.245	1.116	0.301
*LDAH*	3.759	0.038	0.862	0.362	0.503	0.485
***MRPS27***	**2.798**	**0.081**	**0.007**	**0.936**	**0.335**	**0.568**
*EEF1*	11.209	<0.001	3.890	0.060	1.608	0.217
*HMBS*	1.424	0.260	0.038	0.847	2.277	0.144
***MRPS30***	**2.488**	**0.104**	**0.007**	**0.932**	**0.061**	**0.806**
***RPL5***	**4.102**	**0.029**	**0.964**	**0.336**	**0.032**	**0.860**
*TFRC*	1.854	0.178	0.903	0.351	0.808	0.378
**Normalization Factor**	**3.109**	**0.063**	**0.034**	**0.856**	**0.075**	**0.786**

Stable genes and normalization factor are marked in bold.

## Discussion

Analyses of gene expression have optimized the selection of traits of economic interest in animal breeding. RT-qPCR has been the method of choice and recommended for RG analyses in terms that it has been used by many studies in different species, tissues and treatments [11–16, 18, 27].

Recommended values of amplification efficiency were observed for all analyzed primers in this study ranging from 94 to 109%, and R^2^ values above 0.99. Annealing temperature between 60 and 64°C was efficient for amplification of all genes (Table 2). These efficiency values and temperatures differ from those proposed by NASCIMENTO et al., (2015) which used the same primers in muscle tissues from *G*. *gallus* under different treatments. This is due to different conditions in the experimental design and equipment used, highlighting the importance of performing efficiency tests for each experiment.

According to our results, *MRPS27* gene was ranked as the most stable on General group, not considering Genetic group, Sex and Environment factors. In contrast to General group, considering the other conditions (Peloco, Caneluda, Cobb 500^®^, Male, Female, Heat stress, thermal comfort), the most stable genes varied between *RPL5* (Peloco), *HMBS* (Cobb 500^®^ and Heat stress) and *EEF1* (Caneluda). Besides the General group, *MRPS27* gene was ranked as the most stable in terms of thermal comfort, males and females.

The evaluated tools presented a good correlation between each other considering the most stable RG, except for Bestkeeper and Comparative Ct, which displayed greater discrepancy (28% of correlation), contrary to results obtained by NASCIMENTO et al., (2015). These tools use the standard deviation as stability parameter, however, Comparative Ct considers values transformed by ΔCt method and Bestkeeper uses the real values of Ct [7,20,22]. The most correlated tools were SLqPCR and NormFinder (95%). A high correlation between these two tools was expected as they are the most used for analyzing the stability of reference genes, and both perform more specific calculations to determine the most stable RG.

Interestingly, for most of the tools (SLqPCR, NormFinder and Bestkeeper) *ACTA1* gene was above the sixth position in the overall ranking, however, on Comparative Ct (ΔCt) analysis this same gene was considered as the most stable for all analyzed factors. Comparative Ct method uses VANDESOMPELE et al., (2002) methodology, but without the same accuracy, only by comparing the expression of pairs of genes [22], which may explain the observed variation in RG ranking.

As there are variations among breeds, temperatures and physiology between males and females, it is expected to occur such variations beyond statistical differences of algorithms. *MRPS27* and *RPL5* genes remained the most stable for all factors, however, due to the variation between experimental conditions, it is recommended to use a third RG (*MRPS30*) as a control for target genes normalization. *MRPS27* and *MRPS30* gene encodes the 28S subunit protein and are related to death associated protein 3 (DAP3), while *RPL5* encodes a small protein that is a 60S subunit component and is responsible for transporting rRNA 5S to the nucleolus [29].

Results presented in this study point to different RG than those observed by NASCIMENTO et al. (2015), which indicated *HMBS* and *HPRT1* genes, among the same pairs of primer, as the most stable to be used as normalizers for gene expression studies in chickens. However, in agreement to NASCIMENTO et al. (2015), our results showed that *TFRC* gene remains the worst among all tested genes for all categories, except for Cobb 500^®^ animals and heat stress environment, and thus not recommended as normalizer. These different results are due the difference between environments and genetic groups, reinforcing the importance of stability analysis study before any experiment aiming to analyze the expression of target genes for certain treatment.

The importance in choosing ideal reference genes is reflected in the normalization of the expression of interest genes. Furthermore, the use of a non-stable reference gene may negatively affect the results of Fold-Change on the ΔΔCT calculation [27], leading to a misinterpretation of the results. The three reference genes most stable in this study are suitable to normalize gene expression data from different genetic groups of chicken that exhibited divergent behavior, as induced by heat stress. Therefore, these genes must be calculated as a normalization factor as the geometrical mean of selected RG and may be applicable for data normalization in a wide range of gene expression studies using different chicken breeds and environmental factors.

Many RT-qPCR studies have tried to validate reference genes in different species and treatments in livestock, including cattle [11], pigs [12], ovine [13], goats [14], horse [15], birds [10,18,30,31] and fish [16,17], as well as in plants [32,33]. To date, no other study analyzing reference genes under these experimental conditions (environments of heat stress and comfort, different genetic groups and sex) in chickens are known. These results may be used to guide future studies under similar experimental conditions in *G*. *gallus*. Gene expression is the most basic level in which the genotype of an organism leads to phenotype. Furthermore, genes of interest may have a specific pattern of expression under certain experimental condition, giving a wide range of possibilities for functional studies that will allow the identification of suitable reference genes for gene expression normalization.

## Conclusion

Our results indicate RG according to genetic group (Peloco, Caneluda and Cobb 500^®^), sex and environment (heat stress and comfort) effects. For genetic group effect, the recommended RG for Peloco are *RPL5*, *MRPS30* and *MRPS27*; for Cobb 500^®^ are *HMBS*, *RPL5* and *MRPS27*; for Caneluda are *EEF1*, *HMBS* and *HPRT1*. For environment effect, the recommended genes are *HMBS*, *HPRT1* and *LDHA* for thermal comfort; *MRPS27*, *RPL5* and *MRPS30* for heat stress. For sex effect, there was no difference among the most stable genes between males and females, and the recommended genes for both are *MRPS27*, *MRPS30* and *RPL5*.

The *MRPS27*, *RPL5* and *MRPS30* genes remained stable among all analyzed factors and may be recommended for normalization of gene expression data in RT-qPCR studies of breast muscle tissue of chickens from different genetic groups and sex under heat stress conditions. These three genes must be used as a normalization factor calculated by geometric mean to be used in the normalization of target genes.

## Supporting information

S1 Table Ct data of genes. (XLSX)

S2 Table Anova table. (DOCX)
