# Supplementary material for: Influence of heat stress, sex and genetic groups on reference genes stability in muscle tissue of chicken
Source: PLoS One. 2017 May 1;12(5):e0176402. doi: 10.1371/journal.pone.0176402 (PMC5411030; doi:10.1371/journal.pone.0176402)
Supplement: S2 Table — (DOCX) [file pone.0176402.s002.docx]

MRPS30

Df Sum Sq Mean Sq F value Pr(>F)

Raça 2 6.379e-16 3.189e-16 2.488 0.104

Ambiente 1 7.900e-18 7.900e-18 0.061 0.806

Sexo 1 1.000e-18 1.000e-18 0.007 0.932

Raça:Ambiente 2 9.450e-17 4.720e-17 0.369 0.696

Raça:Sexo 2 1.476e-16 7.380e-17 0.576 0.570

Ambiente:Sexo 1 8.200e-18 8.200e-18 0.064 0.803

Raça:Ambiente:Sexo 2 1.206e-16 6.030e-17 0.470 0.630

Residuals 24 3.077e-15 1.282e-16

MRPS27

Df Sum Sq Mean Sq F value Pr(>F)

Raça 2 2.815e-15 1.407e-15 2.798 0.0809 .

Ambiente 1 1.690e-16 1.687e-16 0.335 0.5678

Sexo 1 3.000e-18 3.300e-18 0.007 0.9360

Raça:Ambiente 2 4.660e-16 2.331e-16 0.464 0.6346

Raça:Sexo 2 4.160e-16 2.080e-16 0.414 0.6659

Ambiente:Sexo 1 4.600e-17 4.590e-17 0.091 0.7652

Raça:Ambiente:Sexo 2 3.210e-16 1.607e-16 0.319 0.7296

Residuals 24 1.207e-14 5.030e-16

---

Signif. codes: 0 ‘***’ 0.001 ‘**’ 0.01 ‘*’ 0.05 ‘.’ 0.1 ‘ ’ 1

RPL5

Df Sum Sq Mean Sq F value Pr(>F)

Raça 2 5.017e-12 2.509e-12 4.102 0.0294 *

Ambiente 1 1.900e-14 1.930e-14 0.032 0.8604

Sexo 1 5.900e-13 5.897e-13 0.964 0.3359

Raça:Ambiente 2 2.490e-13 1.247e-13 0.204 0.8169

Raça:Sexo 2 1.550e-13 7.730e-14 0.126 0.8819

Ambiente:Sexo 1 8.300e-14 8.310e-14 0.136 0.7156

Raça:Ambiente:Sexo 2 3.660e-13 1.831e-13 0.299 0.7439

Residuals 24 1.468e-11 6.116e-13

---

Signif. codes: 0 ‘***’ 0.001 ‘**’ 0.01 ‘*’ 0.05 ‘.’ 0.1 ‘ ’ 1

| Normalization Factor  Df Sum Sq Mean Sq F value Pr(>F)  Genetic Group 2 2.018e-14 1.009e-14 3.109 0.063 .  Environment 1 2.400e-16 2.440e-16 0.075 0.786  Sex 1 1.100e-16 1.090e-16 0.034 0.856  Genetic Group:Environment 2 1.830e-15 9.170e-16 0.283 0.756  Genetic Group:Sex 2 1.870e-15 9.350e-16 0.288 0.752  Environment:Sex 1 2.400e-16 2.450e-16 0.075 0.786  Genetic Group:Environment:Sex 2 2.190e-15 1.097e-15 0.338 0.717  Residuals 24 7.791e-14 3.246e-15  ---  Signif. codes: 0 ‘***’ 0.001 ‘**’ 0.01 ‘*’ 0.05 ‘.’ 0.1 ‘ ’ 1 |
| --- |
|  |
| \| > \| \| --- \| |
